# Incidence and outcome of salvage cystectomy after bladder sparing therapy for muscle invasive bladder cancer: a systematic review and meta-analysis

**DOI:** 10.1007/s00345-020-03436-0

**Published:** 2020-09-29

**Authors:** Victor M. Schuettfort, Benjamin Pradere, Fahad Quhal, Hadi Mostafaei, Ekaterina Laukhtina, Keiichiro Mori, Reza Sari Motlagh, Margit Fisch, David D’Andrea, Michael Rink, Paolo Gontero, Francesco Soria, Shahrokh F. Shariat

**Affiliations:** 1 Department of Urology, Comprehensive Cancer Center, Vienna General Hospital, Medical University of Vienna, Währinger Gürtel 18-20, 1090 Vienna, Austria; 2 Department of Urology, University Medical Center Hamburg-Eppendorf, Hamburg, Germany; 3 Department of Urology, University Hospital of Tours, Tours, France; 4 Department of Urology, King Fahad Specialist Hospital, Dammam, Saudi Arabia; 5 Research Center for Evidence Based Medicine, Tabriz University of Medical Sciences, Tabriz, Iran; 6 Institute for Urology and Reproductive Health, Sechenov University, Moscow, Russia; 7 Department of Urology, The Jikei University School of Medicine, Tokyo, Japan; 8 Division of Urology, Department of Surgical Sciences, San Giovanni Battista Hospital, University of Studies of Torino, Turin, Italy; 9 Department of Urology, Weill Cornell Medical College, New York, NY USA; 10 Department of Urology, University of Texas Southwestern, Dallas, TX USA; 11 Karl Landsteiner Institute of Urology and Andrology, Vienna, Austria; 12 European Association of Urology Research Foundation, Arnhem, The Netherlands

**Keywords:** MIBC, Bladder sparing treatment, Bladder, Cancer, Salvage cystectomy, Radiotherapy

## Abstract

**Objective:**

We conducted a systematic review and meta-analysis to assess the available literature regarding the surgical and oncologic outcomes of patients undergoing salvage radical cystectomy (SV-RC) for recurrence or failure of bladder sparing therapy (BST) for muscle-invasive bladder cancer (MIBC).

**Methods:**

We searched MEDLINE (PubMed), EMBASE and Google Scholar databases in May 2020. We included all studies of patients with ≥ cT2N0/xM0 bladder cancer that were eligible for all treatment modalities at the time of treatment decision who underwent BST including radiotherapy (RTX). A meta-analysis was conducted to calculate the pooled rate of several variables associated with an increased need for SV-RC. Study quality and risk of bias were assessed using MINORS criteria.

**Results:**

73 studies comprising 9110 patients were eligible for the meta-analysis. Weighted mean follow-up time was 61.1 months (range 12–144). The pooled rate of non-response to BST and local recurrence after BST, the two primary reasons for SV-RC, was 15.5% and 28.7%, respectively. The pooled rate of SV-RC was 19.2% for studies with a follow-up longer than 5 years. Only three studies provided a thorough report of complication rates after SV-RC. The overall complication rate ranged between 67 and 72% with a 30-day mortality rate of 0–8.8%. The pooled rates of 5 and 10-year disease-free survival after SV-RC were 54.3% and 45.6%, respectively.

**Conclusion:**

Approximately one-fifth of patients treated with BST with a curative intent eventually require SV-RC. This procedure carries a proportionally high rate of complications and is usually accompanied by an incontinent urinary diversion.

**Electronic supplementary material:**

The online version of this article (10.1007/s00345-020-03436-0) contains supplementary material, which is available to authorized users.

## Introduction

Data from large meta-analyses suggest that bladder sparing therapy (BST) could offer equal oncologic outcomes compared to radical cystectomy (RC) in well-selected patients with muscle-invasive bladder cancer (MIBC) [1–3]. The most effective BST strategy consists of maximal transurethral resection of bladder tumor (TURBT) followed by radiochemotherapy (RCT) [4]. This treatment strategy was primarily used for the elderly, frail patients who were ineligible for RC [5–7]. Nowadays, multiple guidelines support the use of BST in the form of a trimodal therapy (TMT) as an alternative to primary RC with curative intent for selected, well-informed and compliant patients, who desire to retain their bladder [4, 8–10]. Despite the failure of all randomized controlled trials due to accrual (e.g. NCT 02716896 and SPARE [11]), the oncological efficacy of TMT seems non-inferior to primary RC in well-selected patients [1–6].

Patients usually would prefer a BST, as it is considered tolerable due to its minimal invasiveness with genuinely manageable toxicity [12, 13]. However, a significant proportion of patients may eventually need a salvage radical cystectomy (SV-RC) due to non-response to BST or local recurrence [14–16]. For an alternative therapy to be embraced, it needs to also ensure that in addition to a low failure rate, the unfortunate cases can be salvaged with minimal adverse events. However, irradiated tissues are usually more fragile and difficult to dissect, leading to an increased risk of complications following SV-RC [17–21]. In addition, the quality of life after SV-RC may not be similar to primary RC, as the optimal patient for TMT has a unifocal cT2 tumor that is fully resectable without carcinoma in situ or hydronephrosis [4]. Patients fulfilling these criteria could also be candidates for a high class, nerve-sparing primary RC with continent urinary diversion.

We, therefore, conducted a systematic review to evaluate the surgical and oncologic outcomes following SV-RC after BST with a curative intent. We also performed a meta-analysis to investigate the pooled incidence rate of SV-RC and assessed the variables associated with an increased likelihood of requirement for SV-RC.

## Methods

### Search strategy for identification of studies

This meta-analysis was carried out based on the guidelines of the Preferred Reporting Items for Meta-Analyses of Observational Studies in Epidemiology Statement and registered on PROSPERO (ID: CRD42020187685) [22]. In May 2020, a literature search on MEDLINE (PubMed), EMBASE and Google Scholar databases was performed using a Boolean operator and the keywords: *salvage cystectomy, rescue surgery, radiotherapy, trimodal therapy, bladder sparing therapy, multimodal therapy, bladder preservation, radiotherapy, radiochemotherapy, pelvic radiation* and *muscle invasive bladder cancer*. Additionally, reference lists of the retrieved articles were analyzed to identify further studies. Studies lacking original patient data, abstracts, and non-English articles were excluded. The primary outcome of interest was the pooled rate of SV-RC, as well as the surgical and oncologic outcomes of patients treated with SV-RC for BST failure. Secondary outcomes of interest were variables associated with a previously reported increased likelihood for SV-RC [23].

### Inclusion criteria

We included all studies of patients with ≥ cT2N0/xM0 bladder cancer who underwent BST which included radiotherapy (RTX). Patients had to be eligible for all treatment modalities at the time of first-line treatment decision. The choice of treatment modality was usually based on patients and/or physicians’ preferences as well as participation in clinical trials. Studies that focused explicitly on elderly patients or patients unfit for surgery (at time of BST) were excluded, as there are limited treatment options for these patients and SV-RC will frequently not be feasible despite being otherwise required. To focus on modern surgical and radiation treatment, we only included studies published between January 2000 and May 2020. To analyze a homogeneous cohort, we excluded studies that focused on primary partial cystectomy or lymph node dissection (LND), brachytherapy or only TURBT/chemotherapy as part of BST. Studies reporting the outcome of SV-RC following pelvic radiotherapy for non-bladder cancer malignancies were excluded. Patient cohorts that focused on pure variants of urothelial carcinoma were also excluded, as there is little data on the value of BST in these patients. Studies reporting results of mixed cohorts were included. Repeated publications from the same authors or institutions were excluded, except for publications with updated or new data from the same institution.

### Data extraction

Data were extracted independently by two authors. Titles and abstracts were screened to determine whether they met inclusion criteria. Full-text publications of all studies not primarily excluded were obtained and reviewed to check eligibility. If rates were not reported, they were calculated using the number of included patients as denominator, whenever it was possible. Non-response to BST was defined as a stable or progressive disease within 6 months after the initial treatment. Perioperative complications were defined as any complications within 30 days of SV-RC. Early SV-RC was defined as a SV-RC within 6 months of BST. Local recurrence was defined as intravesical recurrence. Missing data were obtained by contacting the authors of the relevant studies, if needed. All discrepancies regarding data extraction were resolved by consensus with co-investigators.

### Quality assessment

As the vast majority of included studies were non-randomized interventional studies, assessment of study quality and risk of bias was performed using the methodological index for non-randomized studies (MINORS) criteria [24]. MINORS is a validated tool, which consist of eight different items for the quality assessment of non-comparative studies. A high risk of bias was attributed for studies with less than 12 points.

### Statistical analysis

If sufficient data were available for evaluation, a meta-analysis of proportions was conducted to calculate the weighted summary overall proportion. These pooled rates represent the average from multiple studies weighted by the inverse of their sampling variances and are presented along with 95% confidence intervals (95% CI) calculated using a normal approximation [25]. The proportion of interest was re-calculated from the relevant numerator (*event*) and denominator (*total cases*), if possible. Depending on the distribution of proportions, either no or logistic transformation was applied [25]. When significant heterogeneity (*p* value of < 0.05 in the Cochrane *Q* test and *I*^2^ > 50%) was observed, a random-effects model (DerSimonian and Laird) was applied. The predominantly small sample sizes and retrospective study design prevented multivariable meta-regression due to inability to properly adjust for the effects of confounders. As it has been shown to be problematic in meta-analyses of proportions, assessment of publication bias using funnel plots was not performed [26]. All analyses were conducted using R Studio, Version 3.6.3.

## Results

**Fig. 1:**
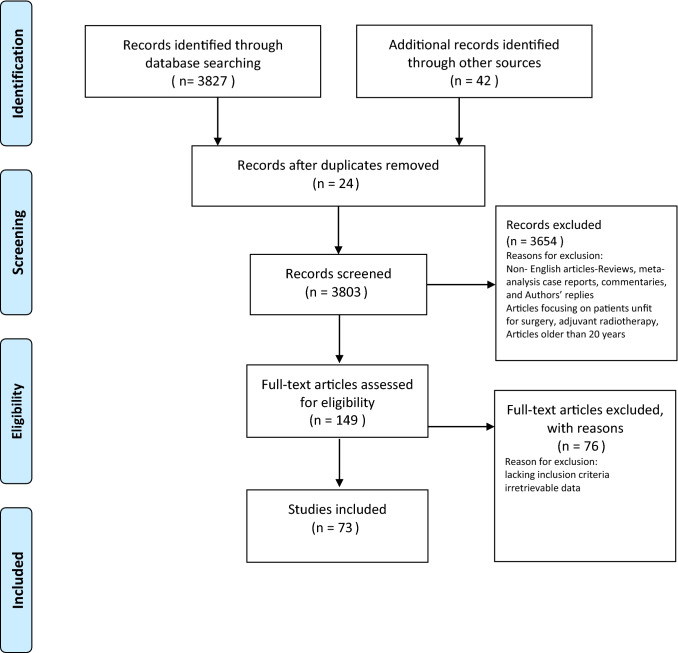
Flow diagram of the study selection procedure for the systematic review and meta-analysis

Overall, we identified 73 studies comprising 9110 patients who underwent primary BST with curative intent according to our inclusion criteria (Fig. 1). The characteristics of included patients and the applied study methodology are shown in Supplementary Table 1 [58–118]. Weighted mean follow-up time was 61.1 months (range 12–144 months).

### Pooled rate of salvage radical cystectomy

**Fig. 2:**
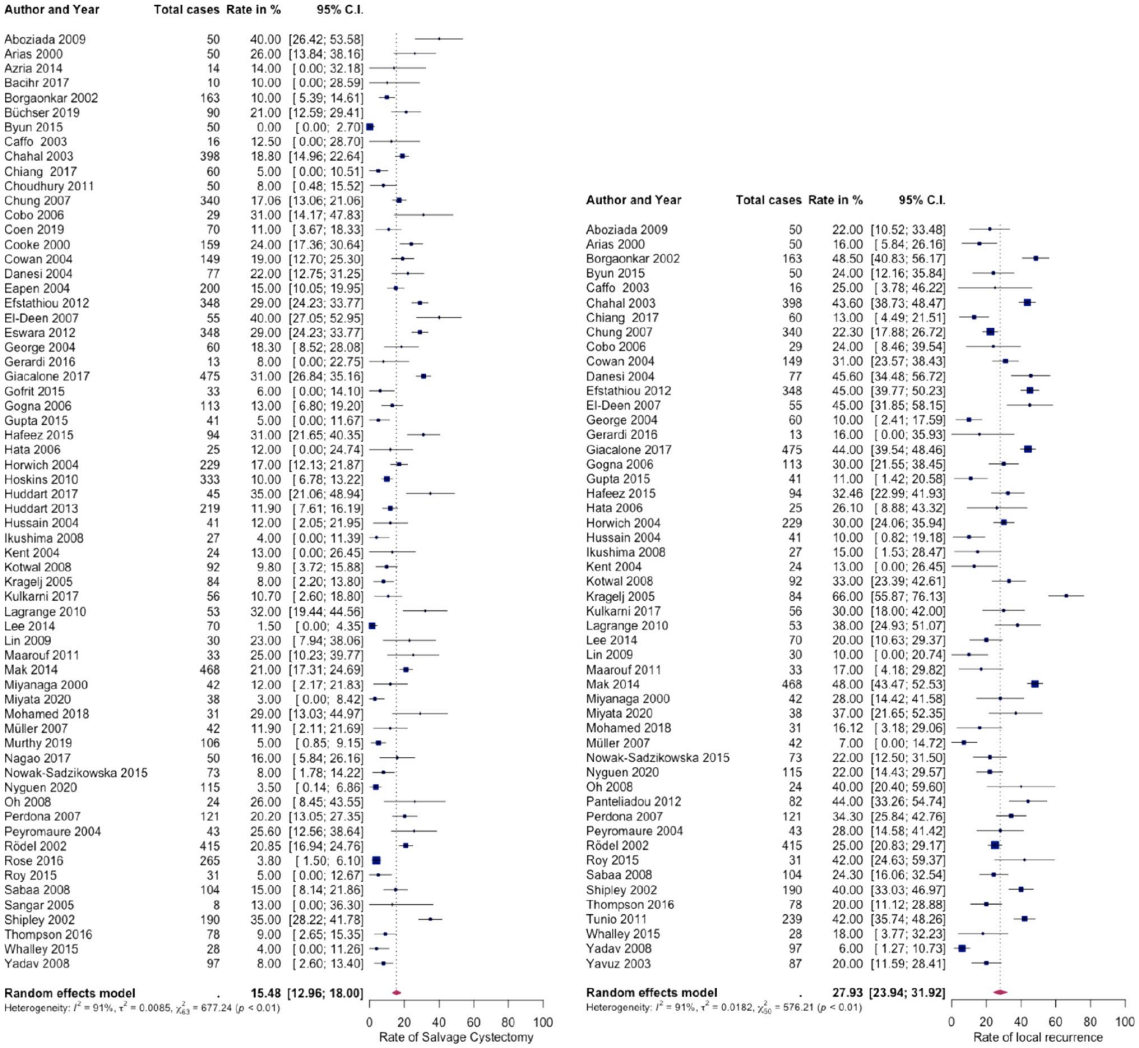
Forest plots showing the pooled rates of salvage cystectomy (left) and local recurrence (right) for studies following bladder sparing treatment of muscle-invasive bladder cancer

The pooled rate of all subsequently necessary SV-RC was 15.5% (95% CI 13.0–18.0, *I*^2^ = 91%, Fig. 2). If only studies with a follow-up longer than 5 years were included, this rate rose to 19.2% (95% CI 15.4–23.0, *I*^2^ = 92%). The proportion of early and late SV-RC was equally balanced (55.7% vs. 44.3%). Pooled rate of incontinent urinary diversion was 91.1% for all reported cases (95% CI 76.1–97.0, *I*^2^ = 68%, logistic transformation applied). In the series of Chahal et al. which compared the outcome of SV-RC and primary RC, 8.3% in the primary RC group had a continent urinary diversion vs. only 3.5% in the SV-RC group. The pooled rate of 5- and 10-year DFS after SV-RC was 54.3% (95% CI 48.6–60.1, *I*^2^ = 79%) and 45.6% (95% CI 41.6–49.6, *I*^2^ = 0%, fixed effect model), respectively.

### Complications rates of SV-RC

Only three studies provided a thorough report of complications after SV-RC, while two further studies provided partial reports. Of these, four studies reported the overall complication rate, which ranged between 67 and 72.2% [27–30]. Two study groups found that the overall complication rates were not significantly worse for SV-RC and primary RC (Chahal et al. [29]: 75% vs 71.4%, Iwai et al. [28]: 67% vs. 57%). The rate of major complications was found to be higher following SV-RC compared to primary RC (22% vs. 12%) [28]. Chahal et al. reported that the perioperative complications rate was lower for primary RC in comparison to SV-RC (37% vs 47.1%) [29]. With respect to specific complications, Iwai et al. report a higher rate of overall urinary anastomosis-related complications (11% vs. 2%, *p* = 0.007) and major gastrointestinal complications (14% vs. 4%, *p* = 0.02) in SV-RC vs. primary RC patients [28]. Similar, Chahal et al. reported a higher rate of bowel leakage following SV-RC, in comparison to patients treated with primary RC (8.7% vs 3%) [29].

30-day mortality rates for SV-RC-treated patients were generally low (Supplementary Table 2), but were higher than those for primary RC (30 day mortality 8.8% vs 3.1%; 3-month mortality: 19.2% vs 14.5%) [29]. Compared to primary RC, SV-RC, therefore, seems to be associated with an increased risk of both complications and perioperative mortality.

### Variables associated with an increased rate of SV-RC

No study focused on analyzing variables that could predict the need for SV-RC. Less than 2% of the patients underwent SV-RC due to non-oncologic reasons [14, 15, 31, 32]. Local recurrence and non-response to BST were found to be the primary reasons for SV-RC. Pooled rate of local recurrence was 27.9% (95% CI 23.9–31.9, *I*^2^ = 92%), this increased to 32.9% for studies with longer than 5 years follow-up (95% CI 27.0–38.7, *I*^2^ = 91%, Fig. 2). In our study, pooled rate of complete response to BST was 75.1% (95% CI 70.8–79.4, *I*^2^ = 95%), while pooled rate for non-response (stable or progressive disease) was 15.5% (95% CI 11.9–19.0, *I*^2^ = 93%). Several studies reported that a significant proportion of patients were not salvageable after BST treatment failure (12–52%) [14, 33, 34].

Two large series noted that in patients with an incomplete primary TURBT before BST, the rate of SV-RC was higher compared to the rest of the cohort (e.g. 42% vs. 22%) [32, 35]. In our study, weighted pooled rate of incomplete TURBT before BST was 38.6% (95% CI 27.9–49.3, *I*^2^ = 99%). Patients undergoing SV-RC had a higher proportion of cT3 and cT4 diseases compared to those who did not need a SV-RC (43% vs. 30%, *p* = 0.007) [35].

### Quality assessment

In terms of methodologic quality, MINORS scores ranged from 7 to 16 (mean 12.7, median 12). Overall, 80.8% (59/73) of all studies included into the meta-analysis were, therefore, found to be without a risk of bias.

## Discussion

The prerequisite criteria for BST to be considered as an adequate alternative to primary RC in patients with MIBC need to be addressed. It is obvious that the equivalence of BST to primary RC, the standard of care in this disease space, can only be assessed in a prospective randomized controlled clinical trial. Since such a study is nearly impossible, due to the patients and physicians’ lack of enthusiasm, we have attempted to investigate the criteria that would allow BST to be accepted as an alternative to primary RC (i.e. low failure rates and a salvage strategy after treatment failure that offers an acceptable morbidity and mortality). We found that one in five patients treated with primary BST with a curative intent subsequently required SV-RC. However, these patients may have represented only a proportion of failures, as not all patients will be eligible for SV-RC. Failure of BST and subsequent time delay might deprive patients of other treatments alternatives [14, 33, 34]. Nevertheless, reported bladder preservation rates of 70–85% seem to be encouraging, especially as further developments in BST, like a tretramodale approach or immunotherapy, might improve treatment outcomes and subsequently reduce the likelihood of subsequent SV-RC [36].

Despite the significant rate of failure in BST leading to subsequent SV-RC, only 3 out of 73 studies thoroughly reported the outcomes of SV-RC. Even with advances in intra- and perioperative care, SV-RC remains a challenging surgery. Indeed, it leads to overall complication rates ranging between 67 and 72% [27–30]. If standardized definitions are applied and a meticulous workup of all complications is performed, these rates are very likely to increase even more [37]. While a non-randomized comparison is not without bias, modern primary RC shows much lower complication rates [38, 39]. Mortality rates of SV-RC were found to be low in our systematic review, but still data suggest worse early and 3 months survival rates following SV-RC in comparison to primary RC [29, 40, 41]. Patients undergoing SV-RC lose their opportunity for a high-quality primary RC (i.e. nerve sparing with a continent urinary diversion). While it still remains unclear if one form urinary diversion is superior to another in terms of quality of life, it is believed that most patients will favor a continent urinary diversion [42]. SV-RC, however, usually entails an incontinent urinary diversion due to irradiation damage to the pelvic tissue. Even though we did not find any data on the use of nerve-sparing SV-RC, it seems unlikely that this is possible, let alone be successful. While a successful BST seems to have little effect on quality of life, a non-nerve sparing approach and an incontinent urinary diversion following SV-RC may potentially decrease the quality of life after failure of BST [43]. This needs to be addressed in the counselling of MIBC patients regarding the risks, benefits and alternatives to primary RC. Patients also need to be counselled on the high local recurrence rate following BST, which necessitates a lifelong follow-up and further invasive procedures, thus increasing morbidity and health care costs [44–46].

Predictors of SV-RC need to be investigated to improve patient selection. In agreement with the literature on oncologic outcome after BST, our findings suggest that patients with multifocal tumors, hydronephrosis non-organ confined disease, incomplete TURBT or carcinoma in situ should not be selected for BST [12]. Despite intensified research, there is a persistent lack of clinical useful biomarkers for patient selection, as all reported results are still exploratory [47–53]. So far, as seen in patients undergoing primary RC, there remains a substantial risk for understaging even in patients seemingly ideal for BST [54–56]. As LND remains the most accurate method for complete pathological staging and thus for detection of more aggressive tumors while simultaneously treating micrometastases, neoadjuvant chemotherapy followed by RC and LND is likely to remain the gold standard for treatment of MIBC in cisplatin and surgically fit patients, as it allows the best risk stratification [57].

The main limitation of this study is its retrospective design. Due to the differences in staging techniques, tumor subtypes, inclusion criteria and treatment modalities the final cohort analyzed was found to be very heterogeneous. Also, we identified only three studies that thoroughly reported the outcomes after SV-RC, as most studies only briefly mentioned the rates of necessary SV-RC. Despite an acceptable quality of the studies included, prospective trials assessing precisely the risk of SV-RC and the oncologic outcomes are highly needed.

## Conclusion

Approximately one-fifth to one-third of patients treated with BST with a curative intent eventually a SV-RC. This procedure has a proportionally high rate of complications and, while primarily entailing a non-tissue sparing (i.e. nerve-sparing) approach, usually is accompanied by an incontinent urinary diversion. The resulting decreased quality of life needs to be taken into consideration during the counselling of MIBC patients regarding the risks, benefits and alternatives (i.e. BST) to primary RC.

## Electronic supplementary material

Below is the link to the electronic supplementary material.

Supplementary file1 (DOCX 10161 kb)

Supplementary file2 (DOCX 36 kb)

Supplementary file3 (DOCX 13 kb)

## Data Availability

All data and material are available.
