# Supplementary material for: Incidence and outcome of salvage cystectomy after bladder sparing therapy for muscle invasive bladder cancer: a systematic review and meta-analysis
Source: World J Urol. 2020 Sep 29;39(6):1757–68. doi: 10.1007/s00345-020-03436-0 (PMC8217031; doi:10.1007/s00345-020-03436-0)
Supplement: Supplementary file 1 — Supplementary file1 (DOCX 10161 kb) [file 345_2020_3436_MOESM1_ESM.docx]

**Supplementary Figures 3-5**


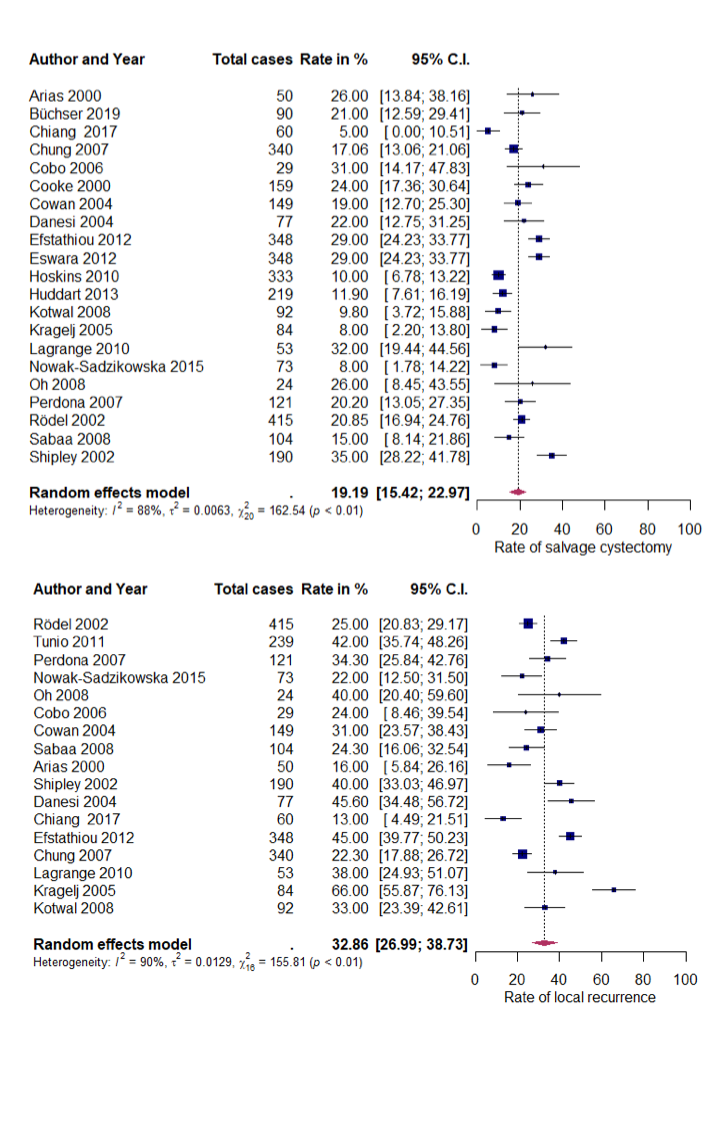


Supplementary Figure 3: Forest plots showing the pooled rates of salvage cystectomy (top) and local recurrence (bottom) for studies with a follow-up longer than five years following bladder sparing treatment of muscle-invasive bladder cancer


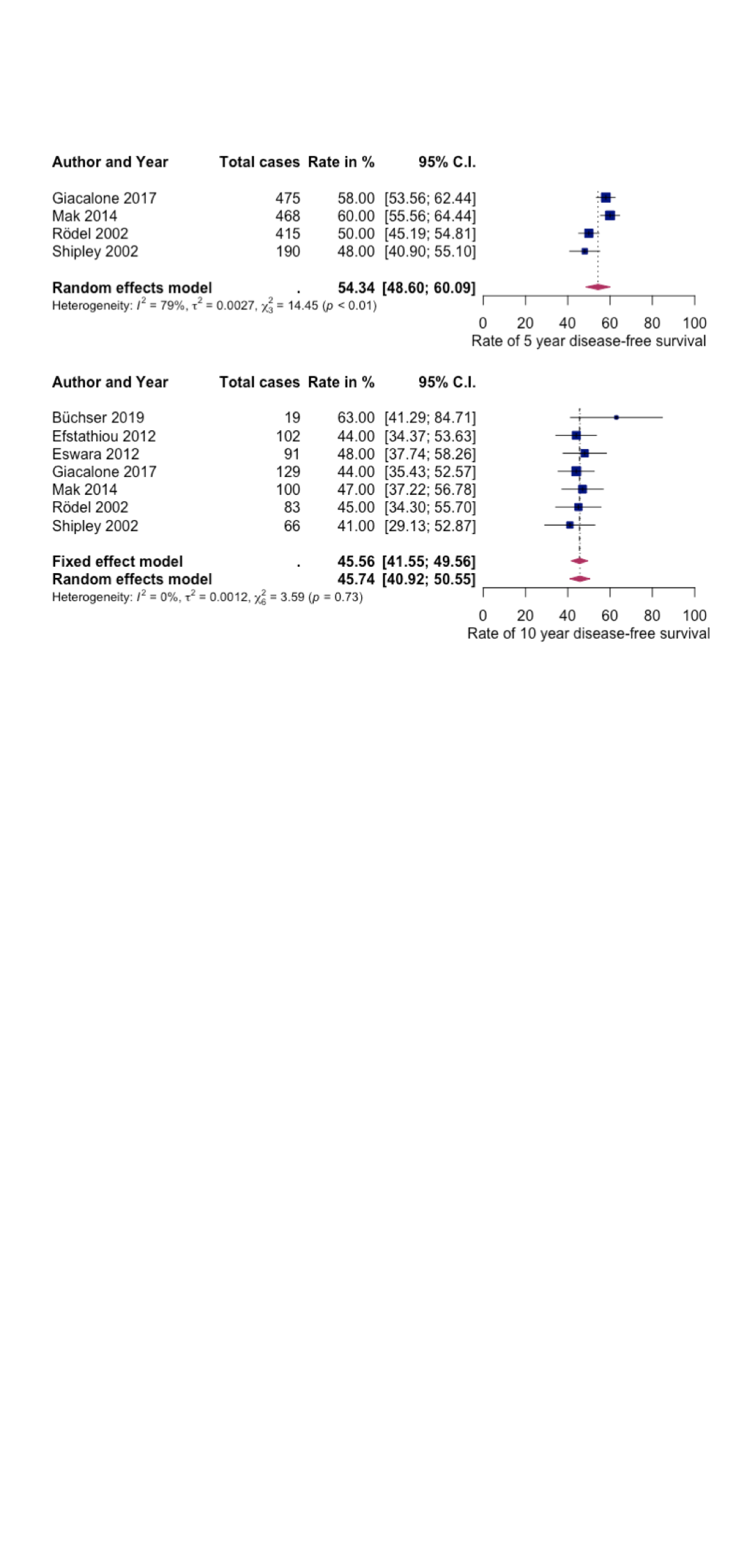


Supplementary Figure 4: Forest plots showing the pooled rate of five and 10-year disease-free survival following salvage cystectomy


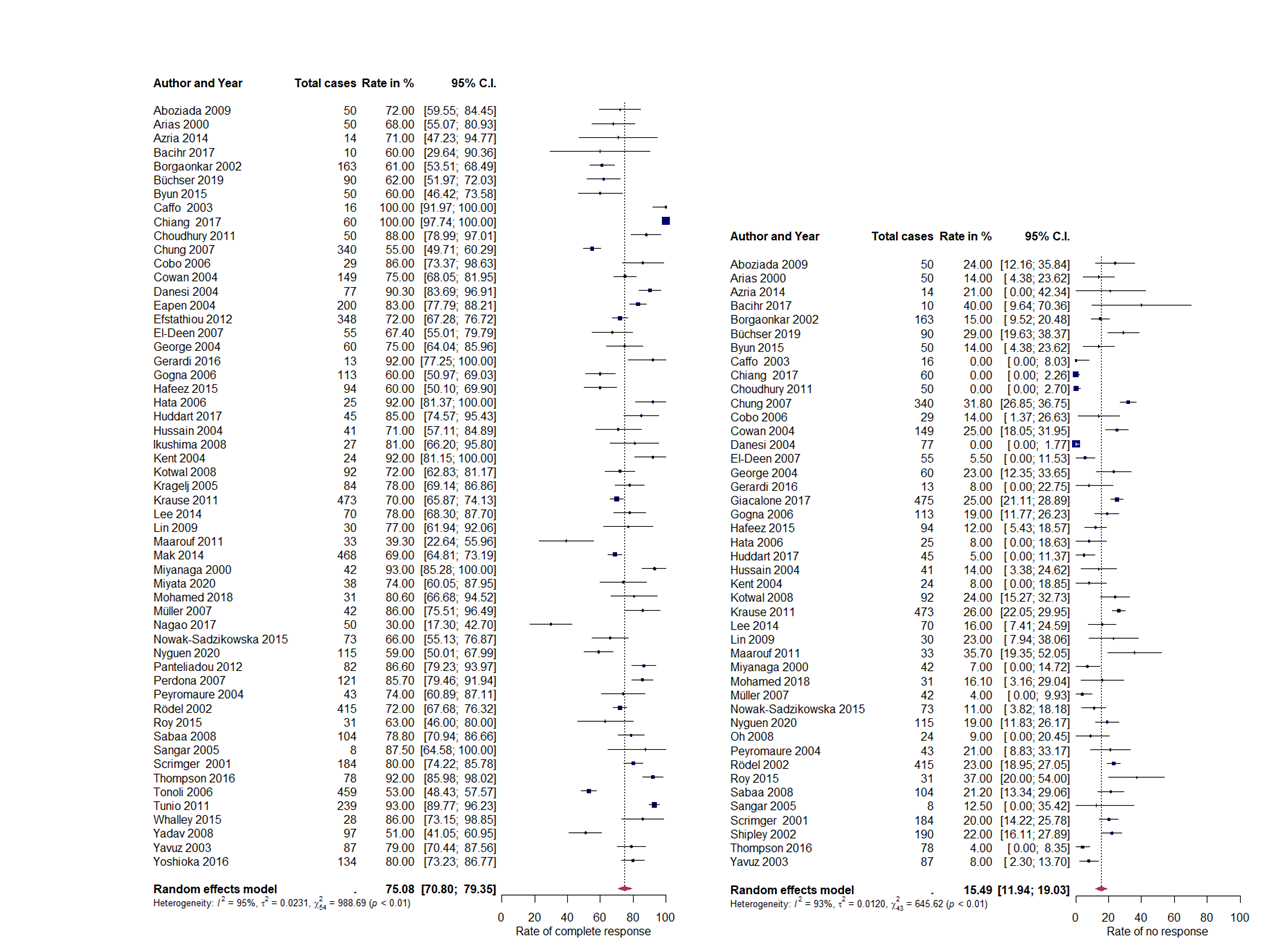


Supplementary Figure 5: Forest plots showing the pooled rates of complete response (left) and non-response (right) to bladder sparing treatment of muscle-invasive bladder cancer (Weighted mean follow-up time: 55.6 months [range: 19.5-71.5 months] for complete response, 53.5 months [range: 12-82.2 months] for non-response)
