# Supplementary material for: Incidence and outcome of salvage cystectomy after bladder sparing therapy for muscle invasive bladder cancer: a systematic review and meta-analysis
Source: World J Urol. 2020 Sep 29;39(6):1757–68. doi: 10.1007/s00345-020-03436-0 (PMC8217031; doi:10.1007/s00345-020-03436-0)
Supplement: Supplementary file 2 — Supplementary file2 (DOCX 36 kb) [file 345_2020_3436_MOESM2_ESM.docx]

SUPPLEMENTARY TABLE 1: CHARACTERISTICS AND OUTCOME OF STUDIES THAT INVESTIGATE BLADDER SPARING TREATMENT FOR MUSCLE-INVASIVE BLADDER CANCER

| **Author +**  **Year** | **Treatment*** | **Stage** | **no. of pat.** | **Age**** | **FU**  ******* | **Incomplete TURBT in %** | **CR in %** | **Non Response in % ****** | **local recurrence rate in %** | **No. immediate SV-RC** | **No. late SV-RC ******* | **SVRC-Rate**  **In %** | **5-year DSS ******** | **10- Year DSS ******** | **Further outcome following SV-RC** |
| --- | --- | --- | --- | --- | --- | --- | --- | --- | --- | --- | --- | --- | --- | --- | --- |
| Miyata 2020 [58] | RCT (40 +25 Gy, platin-based) | cT2-4 N0 M0 | 38 | 80 | 28 | 84 | 74 | NA | 37 | 0 | 0 | 3 | NA | NA | NA |
| Nyguen 2020 [59] | (NAC) + RT or RCT (various regimes) | various | 115 | 79 | 21 | 27 | 59 | 19 | 21.70 | 4 | 0 | 3,5 | NA | NA | NA |
| Coen 2019 [60] | RCT + AC (40+24 GY, Cis +5-FU) | cT2-4a Nx/0 M0 | 70 | NA | 51.6 | NA | 88/78 | 12/22 | NA | 8 | 0 | 11 | NA | NA | NA |
| Murthy 2019 [61] | NAC + RTX/RCT (64 Gy, platin based) | cT1-4 | 106 | 65.5 | 26 | NA | NA | NA | 16 | NA | 5 | NA | NA | NA | NA |
| Büchser 2019 [62] | NAC (MVC) + RCT (60Gy/40,8 + 24 Gy, Cis) | cT2-4Nx M0 | 90 | 63 | 94 | 30 | 62 | 19 | 19 | NA | NA | 21 | NA | 63 | 6/19 alive, 7 dead due to disease |
| Mohamed 2018 [63] | RTX + CTX (patients unfit or refusing RC) | cT2-3 N0 M0 | 31 | NA | 24 | 51.60 | 80.6 | 16.13 | 16.12 | 5 | 4 | 29 | NA | NA | NA |
| Bacihr 2017 [64] | RCT (50 Gy, Everolimus + Cis) | T2-T4a N0M0 | 10 | 78 | 36 | NA | 60 | 40 | NA | 1 | 0 | 10 | NA | NA | NA |
| Chiang 2017 [65] | NAC (Cis + 5-FU or Paclitaxel + Cis or Gem) + RC (45 Gy / 50.4 Gy / 64.8 Gy, Cis +/- Paclitaxe) | cT2-4a N0-1 M0 | 60 | NA | 86.7 | 0 | 100 | 0 | 13.00 | NA | NA | 5 | NA | NA | NA |
| Giacalone 2017 [35] | RCT | cT2-T4a N0 MO | 475 | 67.3 | 54.6 | NA | NA | 25 | 44 | 65 | 64 | 31 | 58 | 44 | for incomplete response the 5-yr and 10-yr DSS were 51% and 32% |
| Huddart 2017 [66] | NAC + RC vs NAC + RCT; (55 / 64 Gy, Gem/Cis) abborted due to recruitment failure | cT2-3 N0 M0 | 45 | 65.7 | NA | NA | 85 | 5 | 66.5 (loco-regional) | 2 | 5 | 35 | NA | NA | 7/20 developed NMIBC in RCT group, 5 were salvaged by SR-RC |
| Nagao 2017 [67] | RCT (43,8 Gy, Cis) | cT2-4 | 50 | 66,5 | 52.3 | NA | 30 | NA | NA | NA | NA | 16 | NA | NA | NA |
| Kulkarni 2017 [3] | NAC (Gem/Cis) + RCT/RT vs. RC (46+20 Gy, Cis) (non- randomized | NA | 56 | 71 | NA | NA | NA | NA | 30.30 | NA | NA | 10.7 | NA | NA | NA |
| Gerardi 2016 [68] | NAC (platin-based) + RCT (56 Gy / 49 Gy / 62 Gy) | cT2 Nx M0 | 13 | 65 | 36 | NA | 92 | 8 | 16 | 9 | 1 | 8 | NA | NA | NA |
| Rose 2016 [69] | RCT (59 Gy, various RS) | cT2-4a N0 M0 | 265 | 75 | 23.4 | NA | NA | NA | NA | NA | NA | 3.8 | NA | NA | NA |
| Thompson 2016 [70] | NAC + RCT vs. RCT (52,5 Grey (hypofractionated) Gem) | NA | 78 | NA | 15.9 | NA | 92 | 4 | 20.00 | NA | NA | 9 | NA | NA | NA |
| Yoshioka 2016 [71] | RCT (50 Gy + 10 Gy, Cis (intra-arterial)) | cT2-4 | 134 | 67 | 31 | NA | 80 | NA | NA | NA | NA | NA | NA | NA | NA |
| Byun 2015 [72] | NAC (mVAC or Gem/Cis) + RCT vs + NAC + RT (63 Gy median) | cT2-4 | 50 | 71.5 | 34 | 76.6 | 60 | 14 | 24 | 0 | 0 | 0 | NA | NA | NA |
| Gofrit 2015 [73] | RCT vs. RC (62 Grey (mean), platin-based + Gem) matched case-cohort study | cT2-4a N0 M0 | 33 | 73 | 35 | NA | NA | NA | NA | 0 | 1 | 6 | NA | NA | NA |
| Gupta 2015 [74] | RT vs RCT (60-70,3 Gy + 56,2 Gy | cT2-4a | 41 | 65,4 | 12 | 0 | NA | NA | 11.10 | NA | NA | 5.12 | NA | NA | NA |
| Hafeez 2015 [75] | NAC (MVAC) + RT (64-68 Gy (median) MMC +5FU) | cT2-4a N0 M0 | 94 | 65 | 39 | NA | 60 | 12 | 32.46 | 10 | 14 | 31 | NA | NA | NA |
| Nowak-Sadzikowska 2015 [76] | NAC (Gem/Cis) + RCT or RT (60 Gy, Cis) | cT2-4a N0 M0 | 73 | 67 | 67 | 67 | 66 | 11 | 22 | 3 | 3 | 8 | NA | NA | NA |
| Roy 2015 [77] | NAC (MVAC) + RT vs NAC + RCT (45+15 Gy, Cis) | cT2-3 N0 M0 | 31 | NA | 12 | 80 | 63 | 37 | 42.00 | 0 | 1 | 5 | NA | NA | NA |
| Whalley 2015 [78] | RTX (66/55Gy (intensity modulated) platin-based) | cT2-4 | 28 | 83 | 24 | 36 | 86 | NA | 18 | NA | 1 | 4 | NA | NA | NA |
| Azria 2014 [79] | RCT (63 Gy, Gem/Cis) | cT2-4 N0 M0 | 14 | 72 | 53 | 0 | 71 | 21 | NA | 2 | 0 | NA | NA | NA | NA |
| Mak 2014 [15] | a total of 6 different BST protocols | cT2-4a | 468 | 66 | 51.6 | 12.20 | 69 | NA | 48 | 62 | 36 | 21 | 60 | 47 | NA |
| Lee 2014 [80] | RCT (59,3 Gy, Cis) | cT2-4 N0 M0 | 70 | 69 | 24.00 | NA | 78 | 16 | 20 | 0 | 1 | 1.5 | NA | NA | NA |
| Huddart 2013 [81] | RCT (55 vs 64 Gy, 5-FU + MMC) whole bladder RT vs. reduced high-dose volume RT | cT2-4a N0 M0 | 219 | 74 | 72.7 | 34 | NA | NA | 29.7 | NA | NA | 11.9 | NA | NA | NA |
| Efstathiou 2012 [32] | RCT +/- NAC/AC (MCV) (40 Gy + boost, Gem/Cis) | cT2-4a | 348 | 66.3 | 92.4 | 33.3 | 72 | NA | 45 | 60 | 42 | 29 | NA | 44 | NA |
| Eswara 2012 [30] | RCT (40+24 Gy, Cis) | cT2-4Nx M0 | 348 | 69.4 | 144 | NA | NA | NA | NA | 50 | 41 | 29 | NA | 48 | 10 Year DFS 38% for immediate 61% for delayed group |
| Panteliadou 2012 [82] | RCT (37,8 / 49,5 Gy, Doxorubicin + Amifostine ) | cT1-4 | 82 | 75 | 21 | NA | 86.6 | NA | 44.00 | NA | NA | NA | NA | NA | NA |
| Zapatero 2012 [83] | NAC (MCV) + RTX / RCT (60/64,8 Gy, Cis) | cT2-4 Nx M0 | 80 | NA | 72 | 28.75 | 74 | NA | NA | 8 | 9 | 21.25 | NA | NA | NA |
| Choudhury 2011 [84] | RCT (52,5 Gy, Gem) | cT2-3 N0 M0 | 50 | 67 | 36 | NA | 88 | 0 | NA | 1 | 4 | 8 | NA | NA | NA |
| Iwai 2011 [28] | RC vs Salvage RCs after BST (40 Gy, Cis) | cT1-4 Nx M0 -1 | 87 | 66 | NA | NA | NA | NA | NA | NA | NA | NA | NA | NA | NA |
| Krause 2011 [85] | RCT or RT (53,8 Gy, platin-based) | NA | 473 | 65.3 | 71.5 | 65.90 | 70 | 26 | NA | 63 | NA | NA | NA | NA | NA |
| Maarouf 2011 [86] | NAC (MVAC) + RTX (40+20 Gy) | cT2-3 Nx M0 | 33 | 56.7 | 12 | 17.9 | 39.3 | 35.7 | 17 | NA | NA | 25 | NA | NA | NA |
| Tunio 2011 [87] | RCT (65 Gy, Cis) whole pelvis vs. bladder only | cT2-4 N0 M0 | 239 | 61,9 | 60 | 23.3-23.6 | 93.1-92.8 | NA | 42.00 | 14 | NA | NA | NA | NA | NA |
| Hoskins 2010 [88] | RTX vs. RCT (55/64 Gy, Carbogen + Nicotinamide) | cT1-4 | 333 | 74 | 57-60 | 37-40 | 76-81 | NA | NA | NA | NA | 10 | NA | NA | NA |
| Lagrange 2010 [89] | RCT (45 +18 Gy, Cis + 5-FU) | cT2-4 | 53 | 68 | 96 | 34 | NA | NA | 38 | 3 | 14 | 32 | NA | NA | NA |
| Aboziada 2009 [90] | RCT (46 +20 Gy, Cis) | cT2-3 N0 M0 | 50 | NA | 18 | 60 | 72 | 24 | 22 | 14 | 6 | 40 | NA | NA | NA |
| Lin 2009 [91] | NAC (Cis + 5-FU +/- Paclitaxel) + RCT (64,8 Gy, Cis +/- Paclitaxel) | cT2-4a N0 M0 | 30 | 66 | 47 | NA | 77 | 23 | 10.00 | 5 | 2 | 23 | NA | NA | NA |
| Ikushima 2008 [92] | RCT (40 Gy, Cis + doxorubicin intra-arterial) | cT2-4 N0-1 M0 | 27 | 71 | 27 | NA | 81 | NA | 15 | 1 | 0 | 4 | NA | NA | NA |
| Kotwal 2008 [93] | RT (55 Gy) vs. RC (non-randomized) | cT1-4 | 92 | 75.3 | 68.1 | NA | 72 | 23.70 | 33 (RT group) | 4 | 5 | 9.8 | NA | NA | NA |
| Oh 2008 [94] | RCT (60 Gy, Gem) | cT2-t3, N0 M0 | 24 | NA | 67.2 | NA | NA | 9 | 30 | 1 | 5 | 26 | NA | NA | NA |
| Sabaa 2008 [95] | RCT (60-65 Gy, Gem/Cis) | cT2-3a N0 M0 | 104 | 54.2 | 71 | NA | 78.8 | 21.2 | 24.3 | 15 | 2 | 15 | NA | NA | NA |
| Yadav 2008 [96] | RC +/- adjuvant RT vs RCT vs RT (40+20 Gy, Cis) | cT2-4a N0 M0 | 97 | 58 | 32 | NA | 51 (RCT) | NA | 6 (RCT) | NA | NA | 8 | NA | NA | NA |
| Chung 2007 [97] | NAC + RTX or RTX or RCT (40/64 Gy, platin-based) | cT1-4 | 340 | 71 | 94.8 | NA | 55 | 31.8 | 22.35 | 36 | 22 | 17.06 | NA | NA | NA |
| El-Deen 2007 [98] | NAC (MVC) + RCT (45 + 19,8 Gy, Cis) | cT2-4 Nx M0 | 55 | 58.8 | 48 | NA | 67.3 | 5.5 | 45 | 18 | 4 | 40 | NA | NA | NA |
| Müller 2007 [99] | RCT (55.8 Gy, Cis +Paclitaxel) | cT1-4 | 42 | 71 | 6 | NA | 86 | 4 | 7.00 | 3 | 2 | 11.9 | NA | NA | NA |
| Perdona 2007 [100] | NAC (MCV) + RTX or RTC (65/45 Gy) | cT2-4 | 121 | 63 | 66 | 19.00 | 85.7 | NA | 34.3 | 10 | 14 | 20.2 | 50 | NA | 50% DFS for all MIBC after SV-RC, 28.5% DFS for non-responding patients |
| Cobo 2006 [31] | NAC (MCV or Gem/Cis) + RCT (64.8 Gy, Cis) | cT2-T3 Nx MO | 29 | 63 | 69 | 31 | 86 | 14 | 24 | 4 | 5 | 31 | NA | NA | 50% of all SV-RC Patients died due to distant metastasis |
| Gogna 2006 [101] | RCT (63/64 Gy, Cis) | cT1-4a | 113 | NA | 23 | 22 | 60 | 19 | 30 | 9 | 6 | 13 | NA | NA | One case of severe post-operative complications (bowel fistula and delayed wound healing) |
| Hata 2006 [102] | RCT (Protontherapy, 41,4 + 33 Gy, MTX + Cis intra-arterial) | cT2-3 N0 M0 | 25 | 72 | 57.6 | NA | 92 | 8 | 26.1 | 2 | 1 | 12 | NA | NA | NA |
| Tonoli 2006 [34] | RTX (60-70 Gy) | cT1-4 N0-Nx M0 | 459 | 67 | 52.8 | 48% | 53 | NA | NA | NA | NA | NA | NA | NA | NA |
| Kragelj 2005 [103] | RCT (64/46 Gy, Vinblastin) | cT1-4a | 84 | 68 | 123.6 | 28% | 78 | NA | 31 | NA | NA | 8 | NA | NA | NA |
| Sangar 2005 [104] | RCT (52.5 Gy, Gem) | cT2-3 N0 MO | 8 | 69.4 | 19.50 | NA | 87,50 | 12,50 | NA | 1 | 0 | 13 | NA | NA | NA |
| Cowan 2004 [105] | RTX whole bladder vs partial bladder (52.5 vs 55Gy) | cT2-3 N0 M0 | 149 | 67.4 | 69.6 | NA | 75 | 25 | 31 | 8 | 20 | 19 | NA | NA | 5-year OS: 54% |
| Danesi 2004 [106] | NAC (MCV) + RCT (69 Gy, Cis + 5-FU) | cT2-4a N0 M0 | 77 | 65.3 | 82.2 | NA | 90.3 | 0 | 45.6 | 5 | 12 | 22 | NA | NA | 9/12 alive at 7 years, 2 dead of UBC, 1 dead to unrelated causes |
| Eapen 2004 [107] | RCT (40+20 Gy, Nedaplatin) | cT1/a-4b N0/1 | 200 | 69 | 34 | 79% | 83 | NA | NA | NA | NA | 15 | NA | NA | NA |
| George 2004 [108] | NAC (MVAC) + RCT (45/64 Gy, platin-based + 5-FU) | cT2-4N0/1 M0 | 60 | NA | 48.5 | NA | 75 | 23 | 10.0 | 6 | 5,0 | 18.3 | NA | NA | NA |
| Horwich 2004 [109] | RTX (60.8/64 Gy) | cT2-3 N0/1 M0 | 229 | 67 |  | NA | NA | NA | 30.0 | NA | NA | 17 | NA | NA | NA |
| Hussain 2004 [110] | RCT (55 Gy, MMC + 5-FU) | cT2-4 | 41 | 68 | 50.7 | NA | 71 | 14 | 10 | 3 | 2 | 12 | NA | NA | NA |
| Kent 2004 [111] | RCT (60 Gy, Gem) | cT2-3 | 24 | 62 | 43 | NA | 92 | 8 | 13 | 1 | 2 | 13 | NA | NA | All patients without evidence of disease post salvage therapy |
| Peyromaure 2004 [27] | RCT (24 Gy, Cis + 5-FU) | cT2 | 43 | 66.3 | 36.3 | NA | 74 | 21 | 28.0 | 2 | 7 | 25.6 | NA | NA | NA |
| Caffo 2003 [112] | RCT (54 Gy, Gem + Cis) | cT2-4 M0 | 16 | 64 | 19 | NA | 100 | 0 | 25 | 0 | 2 | 12.5 | NA | NA | NA |
| Chahal 2003 [29] | RTX (55Gy) or RC | cT1-4 | 398 | 69.4 | NA | NA | NA | NA | 43.6 | NA | NA | 18.8 | NA | NA | NA |
| Yavuz 2003 [113] | RT (superfractionated, 67.5 Gy) | cT1-4 | 87 | 66 | 23 | 2% | 79 | 8 | 20 | NA | 2 | NA | NA | NA | NA |
| Borgaonkar 2002 [114] | RTX (52.5 Gy) | cT1-4 | 163 | 67 | NA | NA | 61 | 15 | 48.5 | 12 | 5 | 10 | NA | NA | Median survival after SV-RC: 12 months |
| Rödel 2002 [14] | RT or RCT (54, platin-based) | cT1-4 | 415 | 67 | 60 | 70.6% | 72 | 23 | 25 | 41 | 42 | 20.85 | 50 | 45 | 5/10 Years DSS for non-responders: 21/18% |
| Shipley 2002 [115] | NAC (MCV) + RCT (40/65 Gy, Cis + 5-FU) | cT2-4a | 190 | NA | 80,4 | 43% | NA | 22 | 40 | 41 | 25 | 35 | 48 | 41 | NA |
| Scrimger 2001 [116] | RT +/- CTX and RC +/- CTX or RTX (56.6 Gy, platin-based) | cT2-4 | 184 | 67 | 19 | NA | 80 | 20 | NA | NA | NA | NA | NA | NA | Median survival after SV-RC: 58 months |
| Arias 2000 [33] | NAC (MVAC) + RCT (45 + 20 Gy, Cis) | cT2-4 N0/1 M0 | 50 | 67.5 | 73 | NA | 68 | 14 | 16 | 10 | 3 | 26 | NA | NA | 8/10 died, 6 due to local or distant recurrence |
| Cooke 2000 [117] | NAC + RCT vs RTX (65 Gy, Cis) | cT2-T4 Nx MO | 159 | 65 | 132 | NA | NA | NA | NA | NA | NA | 24 | NA | NA | Median survival after SV-RC: 15 months, 24% living longer than 5 years, 58% died within 4 years. |
| Miyanaga 2000 [118] | RCT (40/20-30Gy, MTX + Cis intra-arterial) | T2-3 N0 M0 | 42 | NA | 38 | NA | 93 | 7 | 28 | NA | NA | 12 | NA | NA | All salvaged patients without evidence of disease |

RTC= Radiochemotherapy, RTX= Radiotherapy, NAC= neoadjuvant chemotherapy, RC= cystectomy, RS= Radiosensitizer, CTX= Chemotherapy, FU= Follow up in months, Cis= Cisplatin Gem= Gemcitabine, MVC= methotrexate, cisplatin, and vinblastine, MVAC = methotrexate, vinblastine, doxorubicin and cisplatin, MMC= Mitomycin, 5-FU= Fluorouracil

* aside from TURBT ** mean or median *** in months **** after 3-6 months ***** all salvage radical cystectomies performed after 6 months ****** of patients with SV-RC
