# Supplementary material for: Incidence and outcome of salvage cystectomy after bladder sparing therapy for muscle invasive bladder cancer: a systematic review and meta-analysis
Source: World J Urol. 2020 Sep 29;39(6):1757–68. doi: 10.1007/s00345-020-03436-0 (PMC8217031; doi:10.1007/s00345-020-03436-0)
Supplement: Supplementary file 3 — Supplementary file3 (DOCX 13 kb) [file 345_2020_3436_MOESM3_ESM.docx]

SUPPLEMENTARY TABLE 2: OVERVIEW COMPLICATION AND MORTALITY RATES FOLLOWING SALVAGE RADICAL CYSTECTOMY AFTER BLADDER SPARING TREATMENT OF THE BLADDER FOR UROTHELIAL CARCINOMA OF THE BLADDER

|  | **Year** | **Overall complication rate (%)** | **Complication Clavien-Dindo Grades 3-5 (%)** | **Complication Clavien-Dindo. Grades 1-2 (%)** | **30-day mortality (%)** | **3-months mortality (%)** |
| --- | --- | --- | --- | --- | --- | --- |
| Eswara et al. [30] | 2012 | 69 | 16 | NA | NA | 2.2 |
| Iwai et al. [28] | 2011 | 67 | 22 | 55 | 0 | NA |
| Chung et al. [97] | 2007 | NA | NA | NA | 1.7 | NA |
| Peyromaure et al. [27] | 2003 | 72.7 | NA | NA | NA | NA |
| Chahal et al. [29] | 2003 | 75 | NA | NA | 8.8 | 15.7 |
